# The Effect of the Family-Centered Empowerment Model (FCEM) on the Care Burden of the Parents of Children Diagnosed with Cancer

**DOI:** 10.31557/APJCP.2019.20.6.1757

**Published:** 2019

**Authors:** Mahnaz Shoghi, Bahareh Shahbazi, Naimeh Seyedfatemi

**Affiliations:** 1 *Center for Nursing Care Research, Faculty of Nursing and Midwifery,*; 2 *Member of Center for Nursing Care Research, Iran University of Medical Sciences, Tehran, Iran.*

**Keywords:** Family, centered empowerment, cancer, burden of care, family-centered care, children

## Abstract

**Background:**

Families face multiple problems after their children are diagnosed with cancer. This study aims to determine the effect of the Family-Centered Empowerment Model (FCEM) on the care burden of the parents of children, diagnosed with cancer.

**Methods:**

This quasi-experimental study was conducted with 78 parents, having children with cancer. The FCEM was implemented in the intervention group in four stages, namely perceived a threat, self-efficacy, educational participation, and evaluation during four sessions of 20-40 minutes. The control group only received the usual care. The burden of care of the control and intervention groups were measured one month after filling out the initial questionnaire, and one month after the end of the intervention, respectively.

**Results:**

The two groups were similar in terms of demographic variables and level of burden of care. However, a statistically significant difference was observed between the two groups, in terms of the level of burden (p<0.001).

**Conclusion:**

The results of this study showed that the empowerment of parents of children with cancer has an impact on reducing their care burden, and using this empowerment model is recommended to the treatment team, especially nurses.

## Introduction

Cancer is a chronic disease and a leading cause of child and adolescent morbidity and mortality (Berlanga et al., 2016; Siegel et al., 2016). The sudden onset of cancer along with its aggressive long-term treatment and immediate, and often persistent complications, have made cancer a serious issue to deal with (Enskär et al., 2010; Olsson et al., 2015). 

The treatment of children with cancer greatly affects their family members, especially parents, so researchers believe that cancer should be considered a family disease (Woodgate and Degner, 2002; Woodgate and Yanofsky, 2010).

Parents not only constantly reconstruct their roles, functions, interactive and communicative patterns, inside and outside the family, but are also in charge of many caring responsibilities as a result of being the member of the treatment team (Crespo et al., 2016). Clearly, parents not only should tolerate emotional and mental pressure, but also accomplish their medical tasks, parental supervision and all their roles and responsibilities (McGrath, 2002; Grinyer, 2006; Da Silva et al., 2010). Parents of children with cancer usually report more physical and psychological problems, and visit physicians more frequently than others (Young et al., 2002; Rafii et al., 2014).

The researchers showed that a majority of parents experienced care burden, which was associated with the care of their child with chronic illness (Santo et al., 2011; Adelman et al., 2014). The caregiver burden is defined as an objective when it corresponds to the physical and/or mental effects, arising from the act of caring; and as a subjective when it is related to the negative sensation, provoked by the act of caring in the caregiver (Sav et al., 2015).

Understanding disease-related changes among the affected person in the family may help them to feel secure about the future. It also helps them to handle the situation within the family (Wong and Chan, 2006; Maurice-Stam et al., 2008). It is difficult to live with a child with cancer, especially if we have a lack of insight into the disease, and a lack of support from professionals (Sen-Ngam et al., 2005; Saeui et al., 2009).

Parents’ caregiving practice has a significant impact on the condition of children with cancer, whether they are at home or hospitalized. Promoting opportunities to take care of children with cancer, participate in caregiver support groups, as well as discussions between nurses and caregivers can empower caregivers by enhancing their competence in caring. These activities, at the same time also create opportunities for caregivers to share their feelings, and experience the benefits derived from team decision- making (Saeui et al., 2009; Kwak et al., 2011).

Empowerment is a process, which will enable perceived self-efficacy; through this process, the person will be able to analyze the real cause of the problem, they get ready to solve problems by wisdom, sharing knowledge and skills together between health personnel and that person. The process of sharing the knowledge and skills will enhance the person to the perceived self-efficacy, so he/she is able to perform the desired behavior (Borhani et al., 2011; Cortez et al., 2017; Davarpanah et al., 2017).

Chemotherapy is considered one of the prominent stages in cancer treatment. Taking care of the sick child during this stage, is associated with lots of complications. Learning about medicine and their side effects, knowing about treatment conditions and stages, attempting to avoid the child being infected, managing the child’s exhaustion, pain and nutrition, facing unpredictable incidents during treatment and hospitalization may cause many challenges for children’s parents, among which is care burden (Santo et al., 2011; Dambi et al., 2015; Salvador et al., 2015). Family empowerment can decrease the burden level through improving physical and mental function, and increase the self-ability (Karian et al., 1998; Saeui et al., 2009). The family-centered empowerment model (FECM) is designed upon effectiveness of the individual and other family members’ role on the three motivational, psychological (self-esteem, self-control and self-efficacy) and self-problem characteristics (like perceived knowledge, attitude and perceived threat). The main goal of the FECM is to strengthen the family (patient and other members), in order to improve the health level of both the family and the children with cancer (Gibson, 1995; Keshvari et al., 2015). The FECM consists of four steps, including first step: increasing the knowledge level through educational sessions, using educational assistive materials, such as PowerPoint presentations, posters, model and handouts and educational methods as a group discussion, questions and answers (Q and A) and lecture. The second step is self-efficacy improvement; third step is increasing self-esteem through educational participation; and fourth step, includes an evaluation process during the empowerment sessions (Borhani et al., 2011).

One of the ways to enhance the quality of care and minimize unfavorable side effects of chemotherapy, is empowerment and increasing the knowledge of the caregivers/ parents, which can be done, using empowerment programs to promote caregivers’ confidence (Saeui et al., 2009). It also seems that empowerment program may decrease the parental burden of care, while providing care for their children with cancer under chemotherapy. Currently in this setting, the health care team does not utilize an empowerment framework for caregivers of children with cancer. In this approach, sustaining patients’ health will increase opportunities for successful completion of the course of chemotherapy, and diminish its side effects and improve the quality of life, for patients and parents of children with cancer.

This study aimed to determine the effect of FCEM on caregiver burden of parents with children, suffering from cancer. The research question to address here is if the FCEM decrease the care burden of parents of children with cancer, receiving chemotherapy.

## Materials and Methods

This quasi-experimental study with intervention and control groups was conducted in two stages: pre-intervention and post-intervention. 

Assuming that the measure of the effect of FCEM on the parental burden of care (medium effect size) is at least d=10, the sample size in each group was determined to be 34.1, at a confidence level of 95%, and a test power of 80%. With regard to the sample loss, the sample size was finally calculated to be 39, in each of the intervention and control groups.

By referring to the children’s oncology department and clinic, the researchers have identified the parents, meeting the inclusion criteria, using the continuous sampling method. Then, the parents were divided into the two groups of control and intervention, using 2-way blocking method. This study was one-sided blind and the parents did not know about their placement in the intervention or control group. Finally, 78 parents of children, aged 1 to 6 years were enrolled in the study. They were included in the two, intervention (35 mothers and 4 fathers) and control groups (35 mothers and 4 fathers).

To prevent information exchange between the intervention and the control groups, and to homogenize the two groups, parents were selected from two different hospitals.

The inclusion criteria were children’s living with both parents, which have passed at least six months, since the disease onset and child undergoing chemotherapy. The research setting included two specialized educational-medical centers (Ali Asghar) and center for hospitalization of children with cancer in Tehran. 

*Intervention*

The empowerment program for enhancing caregiver competence in taking care of children with cancer, undergoing chemotherapy was developed, by the primary investigator and experts, based on the Alhani empowerment model, consisting of 1) perceived threat, 2) skill and self-efficiency acquisition, 3) gaining self-confidence through educational participation, and 4) evaluation (Borhani et al., 2011). The overall purpose was to empower caregivers by improving their knowledge to develop new skills, which are necessary in taking care of children with cancer. 

The educational content has been assessed and approved by a group of expert nurses. The content of the booklet has been taken from “Children with Cancer: A Guide for Parents- National Cancer Institute, 2014”, which includes 4 sessions (diagnosis, treatment, adaptation strategies, life and care follow-up). In this booklet, each session contains various open questions, through which the researcher used to assess the process, and also for the final evaluation (2014). 

*Intervention Group*

First parents were asked to complete the questionnaire about the parental burden of care; then the FCEM was executed based on the model, including four sessions of 40 minutes, in four consecutive weeks (one session per week, per day). 

The class was set up with parents before each session, and sessions were held based on their availability and convenience. All individual and group sessions were held in an existing study room, beside an oncology department in two hospitals.

The parental burden of care was assessed once again in this group, one month after the last session, using the questionnaire. The researcher was in touch with the participants through telephone and face to face, once a day, to answer their questions in between the four educational sessions.

The empowerment program was delivered as follows: in the first session (40 minutes), using the educational booklet, the researcher talked face to face, to the parents about the types of cancers during childhood, the common signs and symptoms of cancer, diagnostic methods and treatment programs, according to the stage and the condition of the child’s illness; then, these parents were given the essential trainings in those regards. At the end of this session, they were provided with the booklet to study. It must be mentioned that this session was delivered individually, to all the parents in the intervention group.

To implement the second step of empowerment model, the parents were placed in groups of 3-6; in the second session, lasting 40 minutes, they were asked to discuss with the hospital treatment team, concerning the ways of communication and exchange ideas with the team. In this session, they shared their experiences and information about treatment, assessment and controlling the side effects of chemotherapy. The other issues discussed in this session, included the ways to prevent infection, controlling and soothing the pain, vaccination, improving self-control in facing the ill children and their problems, and also the ways to support the other family members. During this session, the researcher tried to lead the discussion indirectly and guide the group to enrich self-efficacy.

Throughout the third session, and in order to implement the third step of empowerment model, every participant was asked to teach one of their family members, who was in charge of taking care of the child, about what they had learnt in the past two sessions, and answer his/ her questions under indirect supervision of the researcher. During this session, any possible problems, such as forgetting the content or transferring incorrect information by the trained parents, were immediately corrected by the researcher. In the fourth session or the final evaluation session, the researcher assessed all the participants in the intervention group by asking questions about the whole content taught, and the issues discussed during the second and third sessions; finally he/ she resolves remaining ambiguities.

It is important to note that the subject matter of the previous session was evaluated by the researcher and at the beginning of each session, by asking one or two questions, and the evaluation was done accordingly. The exclusion criteria among intervention group parents’ was unwilling to continue participation in the study, and absence in educational sessions, even for one, and a family member absentee in the third educational session would lead to their exclusion from the whole study.

*Control group*

The burden of care questionnaire was completed twice, by the parents of this group with a month interval, and without any special interventional implementation. It is essential to mention that the parents of this group received all the usual routine caring in the hospital, during this one month.

*Instrument*

The questionnaire used in this study consisted of two parts. The first part asked about the demographic information on the subjects, including two types: (i) items related to the child, (ii) items related to the parent and other family members. The second part included items related to the assessment of caregiving burden for parents. Zarit burden self-report questionnaire was employed to assess caregiver burden of care. This scale included 22 items about the psychological pressures to assess the caregiving burden for parents. It was developed by Zarit et al. (1980) to measure the level of psychological pressure from the disease. This questionnaire was completed by interviewing family caregivers, where they were asked to answer the questions, using a 5-point scale: 0 (never), 1 (rarely), 2 (sometimes), 3 (often), and 4 (always). The total score of each caregiver determined the degree of caregiving burden. The total score < 30, 31-60, and 61-88 indicated mild, moderate, and heavy burdens, respectively. The minimum and maximum scores of each subject were 0 and 88, respectively. The highest score indicates the greater caregiving burden, and vice versa (Zarit et al., 1980; Schreiner et al., 2006). In addition, the reliability coefficient and internal consistency of the original version of the Zarit burden interview questionnaire were reported as 0.71 (test-retest) and 0.91 (Cronbach’s alpha), respectively. 

Institutional review board approval from Iran University of Medical Sciences was obtained prior to the initiation of the study. Prior to data collection, the principal investigator informed and obtained written consent from participants after screening for eligibility.

To this end, parents of children with cancer who met the inclusion criteria were enrolled in the study, after obtaining their informed consent. To preserve the privacy of each parent and to ensure the confidentiality of their information, they were asked to select a code, and use it as their identity by writing it in a specific blank space on the questionnaire. By doing so, the data collected from each subject could be distinguished from others by these specific codes.

*Statistical Analysis*

Descriptive methods, including tables of relative and absolute frequency distribution, distribution of central indices, and dispersion, as well as statistical tests, including Fisher’s exact test, chi-square test, and independent t-test, were used for data analysis.

## Results

In this study, 70 mothers (89.7%) and 8 fathers (10.3%) were equally divided into the intervention and control groups. Demographic information on the parents and their children are presented in Table 1 and Table 2, respectively. According to the results, the majority of the investigated children in the intervention group were girls (53.8%), and in the control group, the majority were boys (51.3%).

In terms of age, the majority of the investigated children in the intervention (41.0%) and control (53.8%) groups were 3-4 years old. In addition, the majority of the investigated children with cancer in the intervention (64.1%) and control (69.2%) groups had a sibling. According to Table 1, there was no significant between-groups difference in terms of gender, age, and sibling.

The majority of the mothers in the intervention (46.2%) and the control (59.0%) groups were 31-40 years old. Most mothers in the intervention (89.7%) and control (76.9%) groups were homemakers; in addition, the majority of them in the intervention (76.9%) and control (69.2%) groups, had high-school diploma or higher. There was no significant pre-intervention between-groups difference in terms of age, job, and education of mothers. With respect to the fathers, the majority of them in the intervention (74.4%) and control (64.1%) groups were 31-40 years old; in addition, the majority of the fathers in the intervention (92.3%) and control (87.2%) groups were employed. Moreover, most fathers in the intervention (53.8%) and control (64.1%) groups, had high-school diploma or higher. According to Table 2, there was no significant pre-intervention between-groups difference, in terms of age, job, and education of the fathers.

Based on the results, caregiver burden for the majority of parents was weak (48.7%) in the intervention group, and moderate (53.8%) in the control group, before the intervention. The Fisher’s exact test showed no significant between-groups difference, in terms of caregiver burden for parents (p=0.908). According to the results, caregiver burden for the majority of parents was weak (71.8%) in the intervention group, and moderate (56.4%) in the control group, after the intervention. The Fisher’s exact test showed a significant between-groups difference, in terms of caregiver burden for parents (p=0.008) (Table 3). According to the independent t-test results (Table 4), the mean pre-intervention score of caregiver burden for parents in the control and intervention groups, was not statistically significant (p=0.792); however, this difference was significant after the intervention (p<0.001). 

The results of the inter-group comparison showed that the mean parental burden of care, in the control group was significantly higher in the post-test than the pre-test. By contrast, the mean parental burden of care in the intervention group presented a significant reduction in the post-test (Table 5).

## Discussion

This study was conducted to determine the effect of FCEM on caregiver burden of parents with children, suffering from cancer. According to the results, the level of caregiver burden for the intervention group, decreased after the implementation of the FCEM. In other words, parents of children with cancer in the intervention group carried the less caregiver burden, compared to the parents in the control group, after the intervention. The results supported the hypothesis that caregiver’s participation in an empowerment program would significantly increase their competence in caring for children with cancer, undergoing chemotherapy. Steps 1 and 2 of empowerment program were successful in helping caregivers to have a better real perspective, and developing their critical reflection. The empowerment framework improved caregivers’ knowledge and understanding of symptoms and side effects, due to cancer and chemotherapy. These understandings facilitated caregivers’ decisions when considering care alternatives, appropriate to their situation. Step three, was gaining self-confidence through educational participation. According to the model, step four was the evaluation, in this meeting to ensure that parents are able to take care of the child; they were given the opportunity to discuss what they have learned about caring, and communicate with each other.

Few studies have been done on the effect of FCEM on caregivers and family members of children with chronic illness, and most of these studies focused on exuding the programs for the issues on outcomes related to children, suffering from chronic diseases or cancer (Tsay and Hung, 2004; Vahedian Azimi et al., 2010; Cheraghi et al., 2015; Hedayati, 2015; Keshvari et al., 2015; El-Melegy et al., 2016; Cortez et al., 2017). 

**Table 1:** Demographic Information of the Children (N=78)

Characteristics	Intervention Group		Control Group		
	N=39		N=39		
	Number	Frequency	Number	Frequency	
Gender					
Boy	18	46.2	20	51.3	X^2^=0.025
Girl	21	53.8	19	48.7	p=0.651
Age					
(1-2)	10	25.6	4	10.3	t =1.406
(3-4)	16	41	21	53.8	p=0.194
(5-6)	13	33.3	14	35.9	
Having a sibling					
Yes	25	64.1	27	69.2	X^2^=0.231
No	14	35.9	12	30.8	p=0.631

X^2^, chi square –test; t, independent sample t - test; Significance (Sig.): * p – value < 0.05 * *p – value < 0.01*

**Table 2:** Demographic Information of Parents (N=78)

	Intervention		Control		
	Group		Group		
	N=39		N=39		
	(35 mothers and 4 fathers)		(35 mothers and 4 fathers)		
	Number	Percentage	Number	Percentage	
parents age					
(20-30)	17	43.6	12	30.8	
(31-40)	18	46.2	23	59	t=0..150
(41-50)	4	10.3	4	10.3	p=0.512
Employment status of parents					
Unemployed	35	89.7	30	76.9	X^2^=1.308
Employed	4	10.3	9	23.1	p=0.129
Educational attainment of parents					
Blew high school diploma	9	23.1	12	30.8	X^2^=0.586
High-school diploma and higher	30	76.9	27	69.2	p=0.444
Educational attainment of parents					
Below high school diploma	18	46.2	14	35.9	X^2^=0.848
High-school diploma and higher	21	53.8	25	64.1	p=0.357

X^2^, chi square –test; t, independent samples t – test; Significance (Sig.): * p – value < 0.05 * *p – value < 0.01*

**Table 3:** Frequency of Caregiver Burden for the Parents of Children with Cancer, before and after the Intervention in Intervention and Control Groups(N=78)

Caregiver burden	Pre-intervention				After intervention			
	Intervention Group		Control Group		Intervention Group		Control Group	
	N=39		N=39		N=39		N=39	
	Number	Frequency	Number	Frequency	Number	Frequency	Number	Frequency
(88-0)								
Weak	19	48.7	17	43.6	28	71.8	15	38.5
(0-30)								
Moderate	19	48.7	21	53.8	11	28.2	22	56.4
(31-60)								
Severe	1	2.6	1	2.6	0	0	2	5.1
(61-88)								
Sum	39	100	39	100	39	100	39	100
Fisher’s Exact Test	p=0.908				p=0.008			

Significance (Sig.): * p – value < .05

**Table 4:** Mean, and Standard Deviation of Caregiver Burden for the Parents of Children with Cancer before and after the Intervention between Intervention and Control Groups

	Intervention Group		Control Group			
	N=39		N=39			
	Mean	SD	Mean	SD	Independent t-test	
Caregiver burden						
Pre-intervention	33.28	12.65	34.03	12.17	t=0.264 p=0.792	ES: 0.059
After-intervention	24.67	9.01	34.13	11.79	t=3.980 **p<0/001	ES:1.04
Differences between Groups	-8.61	10.2	0.102	6.37	t = 4.547 **p<0/00	ES=0.854

SD, standard deviation; t, independent samples t – test; ES, Effect Size; Significance (Sig.), * p – value < 0.05* p* – value <0.01*

**Table 5:** Mean, and Standard Deviation of Caregiver Burden for the Parents of Children with Cancer before and after the Intervention in Intervention and Control Groups (Inter-Group)

	Intervention group		Control group	
Caregiver Burden	N=39		N=39	
	Mean	SD	Mean	SD
Pre-intervention	33.28	12.65	34.03	12.17
After-intervention	24.67	9/01	34/13	11.79
t-test	t=5.272		t= -0.100	
	df=38		df=38	
	**p<0.00		**P<0/00	

SD, standard deviation; t, Paired samples t-test; df, degree of freedom; Significance (Sig.), * p – value < .05* p* – value < .01*

Some improvement have been shown in studies about the use of a FECM on caregivers and family members of children with chronic illness, such as the quality of life and mental health of families of adolescents with bipolar disorder (Sharif et al., 2016), the relationship of parents with adolescents with a high risk of depression (Connell and Dishion, 2008), following therapeutic protocols for children with cancer by parents at home (Walsh et al., 2014), knowledge of parents with children suffering from cancer about the disease, treatment duration, and end-of-life decisions (Lyon et al., 2013).

The effect of family centered empowerment programs on caregiver burden of parents with children, suffering from chronic diseases has been evaluated in few studies. El-Melegy et al., (2016) reported similar findings. According to them, the FCEM through its three axes, namely motivational, psychological (self-confidence, self-control, and self-efficacy), and capacity of problem solving, reduced caregiver burden. Similar results were reported and showed that the implementation of a kind of family-centered intervention could reduce caregiver burden, and depression symptoms in caregivers (Montgomery et al., 2011). In addition, it showed that the implementation of a kind of family-centered intervention was effective in improving the quality of provided services, utilization of services, and a sense of wellness, and in reducing caregiver burden of patients with Alzheimer’s disease (Kwak et al., 2011). Riasmini et al., (2013) used the above- mentioned model to evaluate caregiver burden on 196 caregivers. The findings of the study suggested positive results by reducing caregiver burden. According to the independent family group model, as a kind of family (or caregiver) empowerment, the health professional (nurse) in the cadre tries to empower the caregivers by discussing the following modules. It seems that the similarity in findings, is due to taking the same steps in performing family empowerment programs enriching plans. Although studies were done on different groups of caregivers and in different societies, they all indicate that care burden in qualified caregivers, is less than other caregivers are. 

Our study showed that the informed participation and motivating parents to gain caring skills is effective in reducing their care burden.

It seems that bringing awareness about various aspects of illness, treatment process, problems caused due to not following the health care providers’ instructions, and the importance of family compliance would increase parent’s perception of the existing threat. Meanwhile, attending in a gathering, where the parents, having the same problem, discuss and talk to group members to solve their problems (problem recognition, aiming, understanding the solutions and planning) would increase their tendency of participation. On the other hand, their attendance in educational discussions focused on their needs, and transferring their information to one of their family member, improves their caring behavior, and decreases their care-giving burden. 

Nurses’ awareness of such caregiving importance can lead to desirable results, concerning nursing services received by these particular families. According to these findings, nurses can employ FCEMs for such patients to take an effective step in the care process, consultation, and participatory problem solving, to reduce the dependence of parents on the medical team, and to enable them to meet their own needs as much as possible. Family-centered care programs will be able to play an essential role in the management of physical and mental health of patients by empowering patients’ families and improving their adherence to the therapeutic regimens.

Regarding the effectiveness of this model on reducing the caregiver burden for parents of children with cancer, investigating the effect of this program on other aspects of the lives of these parents, such as depression, life style and stress would be helpful. In addition, regarding the effectiveness of this model in reducing parental caregiver burden of children with cancer, another important aspect to investigate is the effect of this program on other individuals’ life, such as siblings, involved in providing care for such children.

*Study Limitation*

There were some limitations in this study. The difference in mental and psychological characteristics, difference in interpersonal interactions with patients, and difference in motives and personalities of the subjects, are factors that could not be controlled by the researcher, but may affect the learning, sense of self-efficacy, self-esteem, and self-control, and subsequently the empowerment. 

Verbal and emotional communication with the researcher and other parents during the empowerment sessions, and the friendly atmosphere present in these sessions may also affect the outcomes of the intervention group.

The low number of fathers was another limitation of this study. According to the law in Iran, presence of fathers in the ward is limited to the visiting times, which made their accessibility problematic. Although it was trying to eliminate this problem by going to the clinic, and as a consequence few fathers participated in this study. It is necessary to mention that one of the other limitations was the differences between the atmospheres of the two hospitals, where the study was conducted.

In conclusion, based on the results, implementing family-based empowerment programs help parents of children with cancer recognize their deficiencies, and feel enough power in order to change their situation, and this feeling of ability is achieved through obtaining information, receiving support and promoting life skills. In this study, regarding family-based empowerment program conducted with parents of children with cancer, and also the positive results gained, it is recommended to implement this program at a more extensive level with better facilities, for these parents and their family members.
